# Taxonomic variability and functional stability across Oregon coastal subsurface microbiomes

**DOI:** 10.1038/s42003-024-07384-y

**Published:** 2024-12-19

**Authors:** Hengameh H. Soufi, Robert Porch, Masha V. Korchagina, Joseph A. Abrams, Jared S. Schnider, Ben D. Carr, Mark A. Williams, Stilianos Louca

**Affiliations:** 1 Department of Biology, University of Oregon, Eugene, OR USA; 2 Institute of Ecology and Evolution, University of Oregon, Eugene, OR USA

**Keywords:** Microbial ecology, Metagenomics, Biogeography

## Abstract

The factors shaping microbial communities in marine subsurface sediments remain poorly understood. Here, we analyzed the microbiome of subsurface sediments within a depth range of 1.6–1.9 m, at 10 locations along the Oregon coast. We used metagenomics to reconstruct the functional structure and 16S rRNA gene amplicon sequencing to estimate the taxonomic composition of microbial communities, accompanied by physicochemical measurements. Functional community structure, in terms of the proportions of various gene groups, was remarkably stable across samples, despite the latter covering a region spanning over 300 km. In contrast, taxonomic composition was highly variable, especially at the level of amplicon sequence variants (ASVs) and operational taxonomic units (OTUs). Mantel correlation tests between compositional dissimilarities and geographic distances revealed only a moderate influence of distance on composition. Regression models predicting taxonomic dissimilarities and considering up to 20 physicochemical variables as predictors, almost always failed to select a significant predictor, suggesting that variation in local conditions does not explain the high taxonomic variability. Permutation null models of community assembly revealed that taxa tend to strongly segregate, i.e., exclude each other. We conclude that biological interactions are important drivers of taxonomic variation in subsurface sediments, and that this variation can decouple from functional structure.

## Abstract (web summary)

Metagenomic and 16S rRNA amplicon sequencing of coastal subsurface microbiomes reveals a decoupling between a stable functional structure and highly variable taxonomic composition and also sheds light on the processes driving taxonomic variability.

## Introduction

It is becoming increasingly apparent that subsurface marine sediments, particularly in coastal regions, harbor an enormous number of microorganisms^1–3^. These microorganisms play a major role in organic carbon deposition rates, nutrient cycling and global methane fluxes^4,5^. Yet, the factors shaping microbial communities in subsurface marine sediments remain poorly understood, largely due to sampling difficulties. Most previous studies explored the vertical distribution profiles of microbial taxa and genes along sediment columns, and focused on the factors shaping these vertical profiles^6–10^ (but see ref. ^11^ for a taxonomic survey of subsurface sediments across geographic locations). Such studies have repeatedly confirmed the important role that redox conditions and thermodynamics play in the vertical distribution of microbial metabolic functions across the sediment column^12,13^. However, the relationship between function and taxonomic composition at the community level is less understood. For example, it is unclear whether a community’s metabolic functions are largely decoupled from its taxonomic composition within functional groups. Generally, such a decoupling can occur if the mechanisms that constrain function, such as reaction stoichiometry, resource limitation and physical transport bottlenecks, are separate from the mechanisms controlling which particular taxa get to perform each function^14–16^. A decoupling between taxonomic composition and function implies that taxonomic changes need not necessarily affect ecosystem processes, such as nutrient and energy fluxes, which has implications for how we interpret microbiome surveys and biodiversity trends^17,18^. For example, taxonomic shifts caused by temperature or pH changes due to long-term environmental trends need not a priori have any major impact on ecosystem processes. Reciprocally, such a decoupling affects strategies for managing ecosystems, since selecting for or against specific taxa alone may have little impact on functions of interest^19^. While such a decoupling has been observed in other microbial systems, notably in bioreactors, host-associated microbiome, and the pelagic ocean^15,20,21^, it has not yet been confirmed in marine sediments and more broadly in subsurface environments. If such a decoupling were to be confirmed in subsurface sediments, this would beg the question of how dispersal, abiotic environmental variables, and biological interactions between organisms influence which taxa get to occupy specific metabolic niches in any given geographic location, and at which lateral spatial scales each of these factors becomes important. For example, microbial dispersal rates in the subsurface were found to be much slower than in most surface environments^22^, thus reducing homogenization across space and potentially enabling greater compositional differences between locations.

**Fig. 1:**
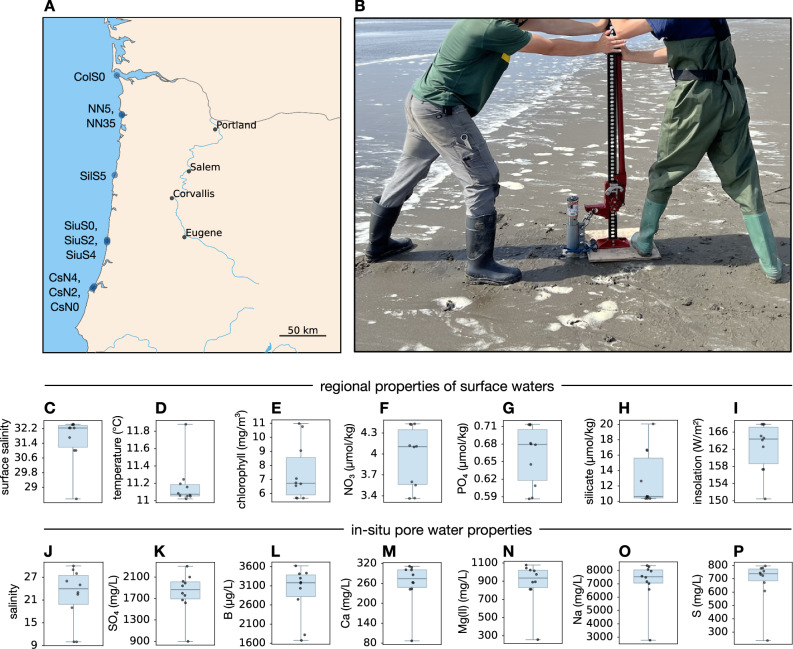
Sample locations and conditions. **A** Locations of subsurface sediment samples examined along the coast of Oregon, USA. **B** Photo of a typical core extraction, here performed South of the Columbia River, Oregon. Photo by HHS. All persons depicted have given their consent to publish this image. **C**−**I** Annual-average regional environmental variables of surface waters, obtained from public gridded datasets and interpolated onto the sampling locations. **J**, **K** Salinity and sulfate concentrations measured in the pore waters collected from the cores. **L**−**P** Concentrations of major elements measured in the pore waters collected from the cores. In each of (**C**−**P**), each scatterpoint represents one sample, boxes span interquartiles, whiskers show the full data range and horizontal line segments show medians. For additional elements and details per sample see Supplementary Data 1.

To address these gaps, here we examined the microbial communities in subsurface intertidal marine sediments within a fixed narrow depth interval (1.6–1.9 m), at 10 different geographic locations along the coast of Oregon, USA. Sampling locations are separated from each other by at least 1.5 km, and cover an area over 300 km across (Fig. 1). We focus in particular on the relationship between taxonomic composition and function, and on elucidating the mechanisms that shape variation in taxonomic community composition across geographic locations, i.e., complementary to the well-established thermodynamic drivers of the vertical distribution of metabolic functions. To this end, we use 16S rRNA gene amplicon sequencing to reconstruct the taxonomic composition of bacterial and archaeal (henceforth simply “prokaryotic”) populations, as well as gene-centric metagenomic sequencing to determine their functional structure, in terms of the proportions of various gene groups. As we describe below, we observed a remarkably similar functional structure in all microbial communities surveyed, despite a highly variable taxonomic composition. Through comparison with multiple environmental variables, as well as through statistical null model tests of community assembly, we further examine various factors that might be driving this taxonomic variation.

## Results and discussion

### Microbial community composition

**Fig. 2:**
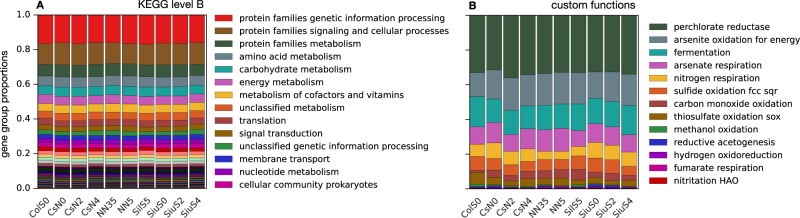
Gene group profiles. **A** Estimated proportions (relative abundances) of genes associated with various KEGG categories at hierarchical level B, based on the average number of metagenomic reads mapped per protein basepair. **B** Estimated proportions of genes associated with various metabolic functions of ecological importance. In both figures, proportions are normalized in each sample such that their sum over all gene groups is 1. For analogous profiles at KEGG hierarchical levels A and C as well as enzyme commission numbers (ECs), see Fig. S2.

To elucidate the functional structure of the surveyed microbial communities, we used gene-centric metagenomic sequencing (sequencing depths in Table S1, collector’s curves in Fig. S1). After assembling reads into contiguous sequences (contigs), predicting and annotating protein-coding genes in the contigs, we determined gene proportions based on reads mapped to contigs. We then classified genes into groups at each functional classification level of the KEGG hierarchy (A to C), with A being the coarsest classification level (e.g., metabolism vs cellular processes), B being a finer level (e.g., energy metabolism vs lipid metabolism) and C being an even finer level (e.g., oxidative phosphorylation vs photosynthesis) corresponding to individual KEGG pathway maps^23,24^. In addition, we grouped genes according to catalyzed reactions based on enzyme commission numbers (ECs^25^), which represent the finest available and meaningful functional classification. We also grouped genes into custom categories involving metabolic functions of general ecological importance in sediments, such as fermentation, methanogenesis or sulfide oxidation. At KEGG level A, profiles were dominated by genes involved in metabolism, followed by genes involved in genetic information processing, and genes involved in environmental information processing (Fig. S2). At KEGG level B, profiles were dominated by genes involved in genetic information processing, genes involved in signaling and cellular processes, and genes involved in metabolism (Fig. 2). In terms of our custom-defined gene groups, we observed a high abundance of genes involved in perchlorate reduction, arsenite oxidation for energy, fermentation, arsenate respiration and nitrogen respiration (Fig. 2B). At each considered KEGG level, all samples had nearly identical functional structure in terms of the proportions of the various gene groups (Fig. 2A and Fig. S2). A similar observation was made for ECs and for our custom gene groups. This suggests that the metabolic pathways and ecological functions of the local microbial communities are of similar importance across samples, and their proportions strongly constrained by similar redox conditions and stoichiometric balances^12,15^. This consistency of functional structure is particularly remarkable given that these samples were obtained along a transect that spans over 300 km. In fact, this transect intersects the deltas of multiple major rivers such as Columbia, Siuslaw, Nehalem and Coos, originating in distinct geographic regions and with discharge rates spanning 3 orders of magnitude (Table S2).

**Fig. 3:**
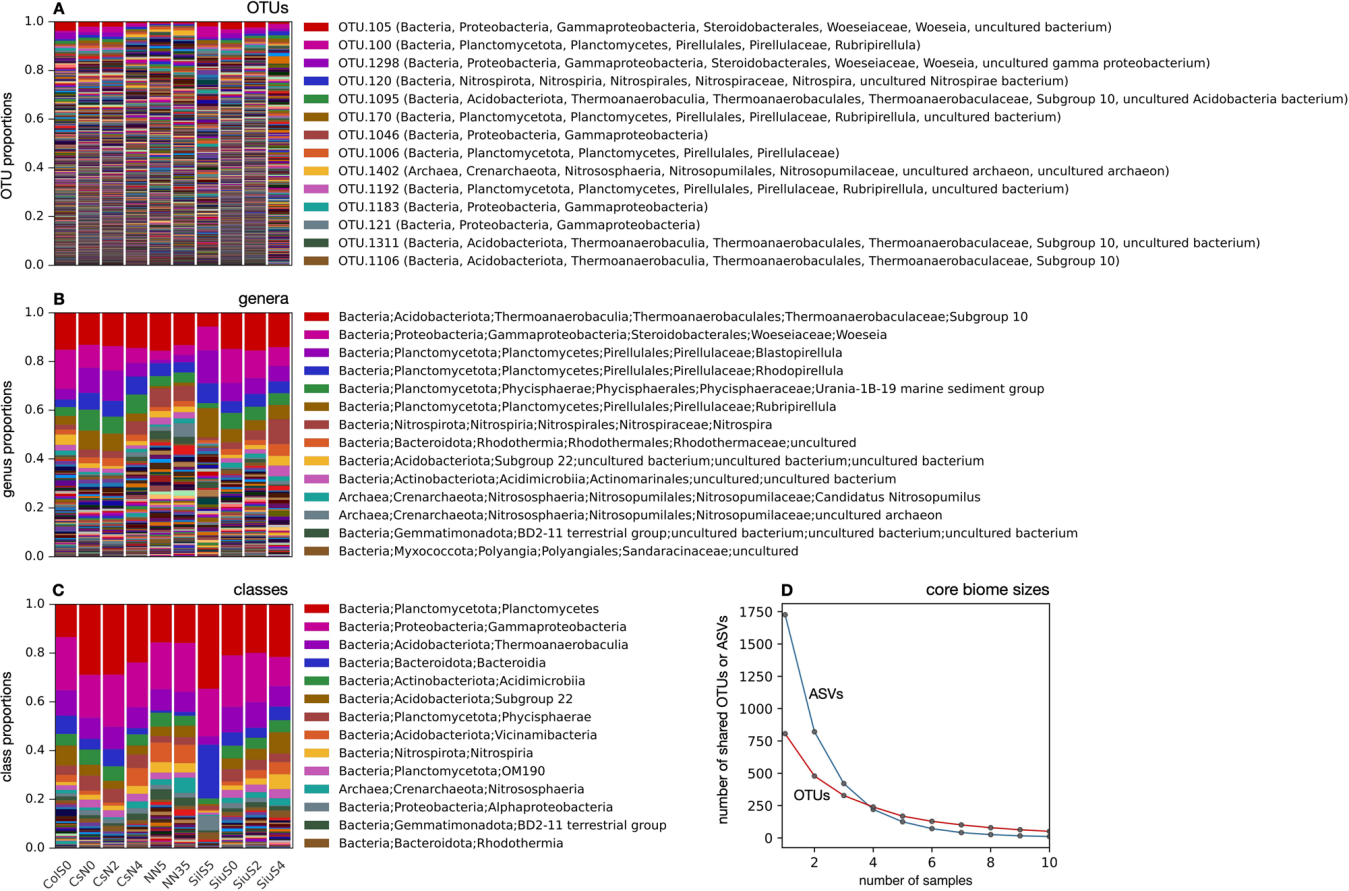
Taxonomic profiles. **A** Proportions (relative abundances) of various prokaryotic Operational Taxonomic Units (OTUs, clustered at 99% similarity), based on 16S rRNA gene amplicon read counts. OTUs are sorted from top to bottom in decreasing average proportion. Only the top few OTUs are listed in the legend for readability. Estimated taxonomic identities of OTUs are written in parentheses. **B**, **C** Similar to A, but showing proportions of genera and classes, respectively. For a similar plot of amplicon sequence variant proportions see Fig. S4. **D** Core biome sizes as a function of the number of samples. For any given number of samples *n*, the curves show the average number of ASVs or OTUs shared by *n* randomly chosen samples.

To elucidate the taxonomic composition of microbial communities, we used 16S rRNA gene amplicon sequencing, with reads either resolved at the level of individual amplicon sequence variants (ASVs)^26^, or clustered into operational taxonomic units (OTUs, 99% similarity)^27,28^ or grouped into higher-order taxa (sequencing depths in Table S1, collector’s curves in Fig. S3). At the genus level, the most abundant taxa were Subgroup 10 in the family Thermoanaerobaculaceae, followed by Woeseia, Blastopirellula and Rhodopirellula (Fig. 3B). At the class level, microbial communities in all samples were dominated by Planctomycetes, Gammaproteobacteria and Thermoanaerobaculia (Fig. 3C). The bulk of the communities, i.e., most of the reads, belonged to taxa whose proportions exhibited high variability across samples. Among the abundant taxa, only a small number had relatively stable proportions across samples. This general taxonomic variability was particularly strong at higher taxonomic resolutions, such as ASV or OTU level. Indeed, nearly all reads were mapped to ASVs and OTUs that exhibited strong fluctuations in their proportions across samples (Fig. 3A and Fig. S4). This observation contrasts the much more stable functional structure across samples discussed earlier. This suggests that while the proportions of various functional groups are similar across all locations, the specific taxa encoding each function are highly variable. As a case in point, the average number of OTUs detected in each sample was 806.2, while the average number of OTUs shared by any two randomly chosen samples was only 478.6 (i.e., down by 40.6%), and the average number of OTUs found in all 10 samples was only 51 (Fig. 3D). This pattern is even stronger at the ASV level: While each sample exhibited on average 1726 ASVs, the number of ASVs shared by any two samples was 821 (i.e., down by 52%), and the number of ASVs shared by all 10 samples was only 11 (Fig. 3D). This means that not only do the proportions of taxa change across samples, many taxa found in one sample can be absent (or at least below detection limit) in another sample. Such a decoupling between functional and taxonomic composition in microbial communities has been reported previously in other environments, notably in bioreactors^18^, human guts^29^, in green macroalga^30^ and bromeliad plants^31^. Explanations previously proposed for this pattern generally invoke functional redundancy, i.e., the existence of multiple taxa capable of similar metabolic functions^15^, combined with specific mechanisms promoting alternative taxa in any given functional group, such as phage-host dynamics^32^, transport-limited metabolic activity^16^ and antibiotic warfare between species^33,34^.

**Fig. 4:**
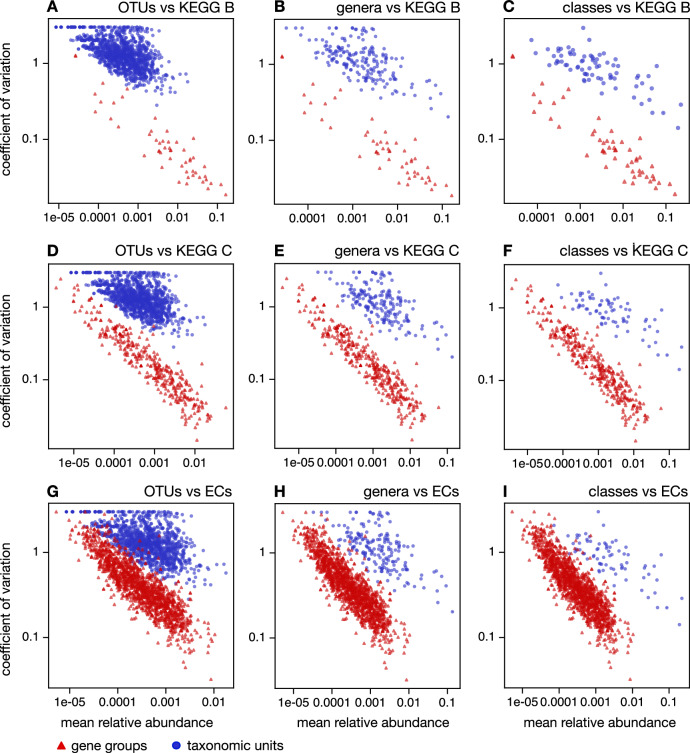
Coefficients of variation. **A** Coefficients of variation of relative abundances (CVs, vertical axis) compared to mean relative abundances (horizontal axis) across samples, for OTUs (blue circles) and KEGG-B gene groups (red triangles). Each point represents one OTU or gene group. **B**, **C** Similar to (**A**), but considering genera and classes, respectively, instead of OTUs. **D**−**F** Similar to (**A**−**C**), but considering gene groups at KEGG level C. **G**−**I** Similar to (**A**−**C**), but considering gene groups at the level of enzyme commission (EC) numbers. In all cases, red triangles represent gene groups while blue circles represent taxonomic units. Observe that OTUs, genera and even classes generally exhibit much greater CVs than gene groups with comparable mean relative abundances.

To more systematically compare the variability of taxonomic and functional composition, we considered the coefficient of variation (CV, standard deviation divided by mean) of the proportions of each taxon and each KEGG gene group across samples. We mention beforehand that in our dataset the CV tends to be smaller for more abundant taxa and for more abundant gene groups (Fig. 4). One obvious technical reason for this is that sampling stochasticity generally decreases with the expected number of matched reads, although less obvious biological reasons may also exist. This correlation between abundance and CV means that comparisons of CVs between different taxonomic levels, or between taxa and gene groups, should account for differences in overall abundances. We thus plotted CVs of various taxa and gene groups as a function of their mean proportion (averaged across samples, Fig. 4). From Fig. 4 it becomes clear that, while some taxa have a lower CV than some gene groups and vice versa, the CVs of taxa tend to be around an order of magnitude smaller than the CVs of gene groups with comparable mean proportions. This observation holds true at all considered taxonomic levels (ASV, OTU, .., phylum) and all considered gene grouping levels (KEGG A, B, C and EC). For example, prokaryotic classes with mean proportions around 0.01 tend to have CVs about 10 times greater than KEGG C gene groups with similar mean proportions (Fig. 4F).

### The role of geographic distance

To assess whether distance-dependent dispersal limitation could explain the observed variation of taxonomic composition, we computed pairwise dissimilarities between samples and performed Mantel tests of Spearman rank correlations between dissimilarities and geographic distances^35^, separately for each considered taxonomic level (ASV, OTU, …, phylum). Dissimilarity metrics that we considered were abundance-based Bray-Curtis, Hellinger and Jaccard^35–37^, which are commonly used in ecology. In contrast to simple correlation tests, Mantel tests are better suited for assessing the significance of correlations between distance matrixes, as they account for interdependencies between matrix entries stemming from the intrinsic data structure. In nearly all of the 21 tests (7 taxonomic levels  × 3 dissimilarity metrics), correlations between dissimilarities and geographic distances were non-significant (details in Table S3). A significant correlation was only observed at the phylum level for Hellinger dissimilarity (correlation 0.35, one-sided *P* = 0.021 based on a permutation test). In fact, if the significance threshold is adjusted to account for multiple hypothesis tests using a Bonferroni correction (*α* = 0.05/21 = 0.0024), none of the correlations are significant. Thus, the influence of geographic distance on taxonomic differences at these spatial scales is not strong enough to be robustly detectable in our dataset. We stress, however, that this does not rule out the existence of dispersal limitation between sampling points. In fact, it is likely that dispersal between sampling points is severely limited over the time scales at which microbial communities typically change^22^, and that correlations between distance and taxonomic composition would be more intense over much shorter distances than those examined here^38^.

### The role of local environmental conditions

To examine the role of environmental conditions as potential drivers of taxonomic variation across samples, we attempted to build linear regression models whose response variables were pairwise dissimilarities of taxon proportions. A separate model was build at each taxonomic level (ASV, OTU, …, phylum) and for each considered dissimilarity metric (Bray-Curtis, Hellinger or Jaccard). As possible predictor variables, we considered pairwise absolute differences in various environmental variables as well as pairwise geographic distances. Environmental variables included annual-average regional oceanographic variables from public gridded databases, such as surface temperature and surface salinity, surface concentrations of chlorophyll, nitrate, phosphate and silicate, as well as concentrations of various elements (B, Ba, Ca, K, Mn, Na, S, Si, Sr), salinity and sulfate concentrations that we measured directly in the collected pore waters (Fig. 1). Note that short-term (e.g., daily or weekly) fluctuations in surface water conditions are unlikely to impact microbial communities in the sampled subsurface layers, due to the slow transport of heat and dissolved substances across sediment columns^16,39,40^ and the fact that microbial cell turnover rates in marine subsurface sediments are generally slow^41–44^. Predictors were selected one-by-one in a stepwise manner, keeping any predictors whose coefficients were statistically significant (*P* < 0.05).

We found that in nearly all cases, i.e., for most taxonomic levels and dissimilarity metrics, none of the considered variables were chosen as model predictors, that is, the majority of coefficients were non-significant (details in Table S4). The only exceptions were models that predicted Hellinger dissimilarities at the phylum, class, family or genus level, where Boron concentration was selected as the sole predictor, achieving a fraction of explained variance during cross-validation (${R}_{{{\rm{cv}}}}^{2}$) between 0.22 and 0.27. The selection of boron as predictor in some cases appears surprising to us, and we are not aware of an obvious mechanism by which boron would influence microbial communities more strongly than other examined factors. We speculate that perhaps boron merely correlates with — and thus acts as a proxy for — other non-measured environmental factors impacting or impacted by microbial communities. For example, boron can be strongly enriched in certain organic compounds in sediments^45^ and is known to interact and bind with clay minerals^46^. If the significance threshold were to be adjusted for the multiple hypothesis tests across taxonomic levels, metrics and candidate predictors (*α* = 0.05/(3 × 7 × 21) = 0.00011), then neither boron nor any other predictor is selected. Thus, much of the taxonomic variation across samples was generally poorly explained by the considered environmental variables, suggesting that this variation is driven mostly by other factors. This finding reflects similar observations in surveys of other environments when functional composition was either constant or separately accounted for, for example in the foliage of bromeliads^31^, in ocean waters^21^ and in soils^47^.

### Null model tests of community assembly

To examine the potential role of biological interactions, such as antagonism or mutualism, in the taxonomic composition of microbial communities, we performed two alternative null model tests commonly encountered in ecology^31,48,49^. One test is based on taxon occurrence (i.e., presence/absence) patterns^50^ and the other test is based on relative abundance patterns^51^. Both tests define a summary statistic that measures the degree to which taxa overlap in their distributions across samples, and a null model from which random composition data can be generated for computing an expectation and statistical significance of the summary statistic. The two summary statistics are henceforth referred to as “CC score” and “MA score”, respectively; precise mathematical definitions and details on the null models are given in the methods. A significantly high CC or MA score means that taxa tend to co-occur more frequently than expected by chance, potentially due to positive interactions, while a significantly low CC or MA score means that taxa tend to segregate, potentially due to negative interactions. Detailed results at each taxonomic level for the two tests are given in Tables S5 and S6. We found that at all CC scores and all MA scores were smaller than expected under the null model, regardless of taxonomic level. All MA scores were statistically significantly low (*P* < 0.05), even when adjusting the significance threshold for multiple hypothesis tests using a Bonferroni correction (*P* < 0.0071). Similarly, nearly all CC scores (except at phylum and class level) were statistically significantly low even after Bonferroni correction (*P* < 0.0071). This strongly suggests that taxa tend to segregate in their distributions. This result is particularly remarkable in view of the fact that the null model considered for the CC scores, known as “fixed-fixed” model, is generally regarded as conservative, i.e., frequently accepting the null hypothesis^50^. These results thus suggest that community assembly is strongly influenced by mutual exclusions between taxa, likely due to biological interactions. Similar null model analyses of microbial communities in bromeliad foliages also revealed significant segregation patterns between OTUs^31^. In both the present study and in the bromeliads, the actual biological mechanisms driving this segregation remain unknown, and may include for example apparent competition driven by phages and antibiotic warfare between bacteria^15,32^. That said, additional experimental evidence, such as direct observations of interactions or experiments manipulating community composition, is needed to confirm these statistical inferences.

### Conclusions

We have presented a systematic examination of the microbial communities in subsurface coastal sediments, along a transect spanning hundreds of kilometers and covering multiple river deltas. Despite these large spatial scales, the functional structure of the communities in terms of the proportions of various gene groups was remarkably constant across all samples, even at the highest resolutions considered (ECs and KEGG level C). In contrast to this stable functional structure, we observed strong variations in taxonomic composition, especially at lower taxonomic levels (ASV, OTU and genus). This taxonomic variability persisted, and in fact became even more evident, when controlling for the overall proportion of taxa and gene groups in a sample, that is, when comparing taxa and gene groups with similar overall proportions. Overall, these results suggest that community-level function in marine subsurface sediments may be decoupled from taxonomic composition within functional groups. In particular, despite the strong environmental filters of metabolic functions across depth^6–10^, additional mechanisms can cause high taxonomic variation within a given layer. Conceptually, this means that one might separate community composition into two perpendicular axes of variation, function on the one hand and taxonomic composition within functional groups on the other hand, with each axis being controlled by separate mechanisms^14,21^. Such a separation, in turn, can guide the development of nested models for microbial community assembly, first modeling function regardless of taxonomic composition^16,52^ and subsequently modeling taxonomic variation separately in each functional group^53^. Here we statistically investigated various alternative factors that might explain the observed taxonomic variation despite strongly constrained functional structure, including geographic distance, several environmental variables and intrinsic biological interactions. We found little evidence that geographic distance and the considered environmental variables drive this variation at the considered spatial scales. Instead, we conclude that biological interactions – primarily antagonistic – likely strongly affect community assembly, and appear to cause a statistically significant segregation of the distributions of various taxa across samples.

## Methods

### Sample collection and measurements

Samples were collected from marine coastal subsurface sediments at 10 different locations across the Oregon coast. An overview of sampling locations is provided in Fig. 1 and in Fig. S1. All samples were collected within the intertidal zone, within a depth range of 1.6–1.9 m. A galvanized steel pipe (6.045 cm diameter) was pushed into the ground using a gasoline-powered post driver, sealed at the top using a Gripper® expanding pipe plug, and subsequently pulled out using a farm jack affixed to the pipe with a Morris coupling. This sampling depth was chosen as the maximum depth that could be practically reached with an 8-foot steel pipe, which in turn was the practical size limit given the transportation means permitted at some locations, the equipment needed for inserting and extracting the pipe, and ultimately our overall budget. While we have no concrete reason to expect our overall conclusions to only be valid for this sampling depth, the generality of our findings can only be fully confirmed by future studies examining alternative depths. Material for DNA extraction was collected in 50 ml centrifuge tubes after breaking the seal created by the gripper plug and sliding out 30 cm of the deeper end of sediment core. Water for chemical analyses was collected from the same cores by centrifuging the collected sediment and transferring the supernatant water to second tubes for storage. All samples were stored on dry ice in the field and subsequently at −80 °C in the laboratory until further processing.

DNA was extracted from the collected sediments using the Qiagen™ DNeasy PowerBiofilm kit following the manufacturer’s protocol. To reduce spurious variance in microbial community compositions merely due to microheterogeneities and due to the coarseness of the core’s depth estimates, 3 extractions were performed from each core from nearby layers and subsequently pooled, roughly equally spaced within the depth range 1.6–1.9 m. Shotgun metagenomic and 16S rRNA gene amplicon sequencing was performed for each of the 10 samples by the Integrated Microbiome Resource (IMR) in Dalhousie, Canada. Specifically, metagenomic libraries were prepared using the Illumina Nextera Flex kit and sequenced using a NextSeq2000 (2 × 150 bp paired ends). 16S rRNA gene amplicon fragments (V4-V5 region) were PCR-amplified using the Phusion Plus polymerase and “universal” bacterial + archaeal primers (515FB = GTGYCAGCMGCCGCGGTAA, 926R = CCGYCAATTYMTTTRAGTTT^54,55^), and sequenced on a MiSeq (2 × 300 bp paired ends).

### Environmental variables

In-situ salinity of pore waters was measured using a refractometer. In-situ sulfate concentrations (mg/L) were measured using a Hach® DR1900 spectrophotometer and the Hach® TNT 865 sulfate kit. Concentrations of boron, barium, calcium, potassium, magnesium (I and II), manganese, sodium, sulfur, silicon and strontium were determined using Inductively Coupled Plasma Optical Emission spectroscopy (ICP-OES) at the Keck laboratory, Oregon State University. Overviews of the main elemental concentrations measured are shown in Fig. 1. For detailed measurements per sample see Supplementary Data 1. Annual average values of regional environmental variables used for model building were determined for each sampling location based on publicly available datasets, accessed on May 21, 2024. Monthly average ocean surface temperatures, surface chlorophyll concentrations and solar insolations were downloaded from the NASA Earth Observations gridded database, spatial grid resolution 0.25° (dataset IDs MYD28M, MY1DMM_CHLORA and CERES_INSOL_M, respectively), and subsequently averaged over the 12 months preceding our sample collections (October 2022−September 2023). Monthly multi-year average ocean surface nitrate, phosphate and silicate concentrations were downloaded from the World Ocean Atlas database release 2023^56^, hosted by the US National Centers for Environmental Information, accession 0270533, spatial grid resolution 1°, and subsequently averaged over all 12 months. Monthly multi-year average ocean surface salinities were downloaded from the World Ocean Atlas database release 2023, spatial grid resolution 0.25°, and subsequently averaged over all 12 months. Gridded data were evaluated at sample locations via bilinear interpolation. If a sample was located inside a grid cell where values were missing on some or all cell corners, interpolation was done using a triangulation of the grid points with non-missing values.

### Analysis of 16S rRNA gene amplicons

On average 11846 16S rRNA gene read pairs were obtained for each sample. Reads were quality-filtered and amplicon sequence variants (ASVs) were inferred and chimera-filtered using the R package dada2 v1.28.0^26^, as follows. Reads were quality-filtered using the dada2 function filterAndTrim, with options “truncLen = (250,200), maxEE=(1,1), truncQ=(0,0), trimLeft=(6,6), minLen = (100,100), maxLen = (100000,100000)”, retaining on average 9327 read pairs per sample. Error model calibration for ASV inference was performed jointly for all samples but separately for forward and reverse reads. Calibration was performed using the dada2 function learnErrors with options “nbases=1e8, randomize=TRUE, MAX_CONSIST=10, errorEstimationFunction=loessErrfun”. Reads were dereplicated using the dada2 function derepFastq. ASVs were inferred from the dereplicated sequences separately for forward and reverse reads, using the dada2 function dada (options “pool=TRUE, selfConsist=FALSE”) and the previously calibrated error models. ASVs from forward and reverse reads were merged using the dada2 function mergePairs with options “minOverlap=12, maxMismatch=0, trimOverhang=TRUE”. Merged ASVs were chimera-filtered using the dada2 function removeBimeraDenovo (option method = 'consensus'). This yielded an ASV table of 4380 chimera-filtered ASVs, accounting for 54116 reads across 10 samples.

ASVs were taxonomically classified based on a comparison to the SILVA database v138.1^57^, using a consensus approach^58^. In total 1 ASV was identified as chloroplast, no ASVs were identified as mitochondria and 64 ASVs could not be classified at any taxonomic level; these ASVs were omitted from subsequent analyses. This left us with 4315 prokaryotic ASVs, accounting for 53477 reads across all samples. To remove species-level redundancies in ASVs, we also clustered ASVs into operational taxonomic units (OTUs) de-novo at 99% similarity^27,28^. Clustering was done using vsearch –cluster_fast with options “–iddef 2 –strand plus”, which yielded 1526 prokaryotic OTUs. Taxonomic identities of OTUs were inherited from their representative (centroid) ASVs. To examine the achieved taxonomic coverage of our samples, we computed collector’s curves (also known as “accumulation” or “rarefaction” curves) of the number of taxa discovered versus the number of reads (Fig. S3). Pairwise dissimilarities between samples in terms of microbial taxonomic composition (ASV and OTU levels) were computed using 3 different metrics, all of which accounted for ASV/OTU abundances: Bray-Curtis, Hellinger and Jaccard^35^. These dissimilarity matrixes were used for Mantel tests and regression analysis, described below.

### Analysis of metagenomes

On average 4,788,369 metagenomic read pairs (~1.2 Gbp) were obtained per sample. Adapters were trimmed from reads using the tool skewer v0.2.2^59^. Reads were then quality-filtered using vsearch v2.22.1^60^ with options “–fastq_ascii 33 –fastq_maxee 0.2 –fastq_truncee 0.2 –fastq_qmax 64 –fastq_maxee_rate 0.002 –fastq_stripleft 0 –fastq_trunclen_keep 10000”, retaining on average 3,164,169 high-quality read pairs per sample. Paired reads from all 10 samples were coassembled into longer contiguous sequences (contigs) using megahit v1.2.9^61^ with option “–min-contig-len 500”. A total of 133,241 contigs were generated, with an average length of 821 bp, a maximum length of 44,434 bp and an N50 of 780.

Gene-centric functional profiles were generated from assembled contigs similar to^58^. Here we thus only provide a brief summary. Contig coverages were computed for each sample by mapping the non-assembled reads to the contigs, then counting the number of reads mapped to each contig with a MAPQ score ≥30 and dividing that number by the contig length. Contig coverages were then normalized in each sample to sum 1, thus yielding contig “proportions”. Protein-coding genes (PCGs) were predicted in the contigs using prodigal v2.6.3 with option “-p meta” and otherwise default options^62^. PCGs were then either mapped to KEGG gene orthologs (KOs) in the KOfam HMM database r105^63^, or mapped to the AsgeneDB amino-acid sequence database of arsenic-metabolism-related genes^64^, or mapped to a custom set of perchlorate reduction genes (*pcrABC*). Only hits with an E-value below 10^−10^ were considered. Proportions of PCGs were computed in each sample by first associating with each PCG the proportion of its host contig, and then normalizing those values in each sample to sum 1; in other words, PCG proportions express the relative abundance of each PCG in a sample compared to all predicted PCGs. The proportion of a given gene in a given sample was estimated by summing the proportions of all proteins mapped to the specific gene. To obtain profiles of the functional potential of each sampled microbial community, genes were assigned to custom functional groups described previously [^58^ Table S3 therein]. KOs were also grouped into standard KEGG categories, at hierarchical levels A, B and C. In addition, KOs were grouped according to their Enzyme Commission (EC) numbers^25^, which correspond to distinct enzymatic functions and provide the highest meaningful resolution of functions. Note that the individual KOs represent level D in the KEGG hierarchy; since KOs are defined based on orthology and not based on function, level D profiles are not strictly speaking functional profiles and are thus not considered here. The proportion of each functional group (or KEGG category or EC) in each sample was computed as the sum of proportions of all associated genes. The following KEGG categories were omitted, as they are not actually defined based on function: “brite hierarchies”, “enzymes with ec numbers”, “not included in pathway or brite”, “poorly characterized”, “general function prediction only”, “others”, “unclassified viral proteins”, “function unknown”.

### Regression analysis and Mantel tests

To examine the potential role of dispersal on microbial community structure, we performed Mantel rank correlation tests^35^, comparing pairwise dissimilarities in community composition to pairwise geographic distances. We considered 3 of the most common dissimilarity metrics, Jaccard, Bray-Curtis and Hellinger^35–37^, calculated at the level of ASVs as well as OTUs. The one-sided statistical significance of Spearman rank correlations was estimated through 1000 random permutations of the dissimilarity matrix’s rows and columns, each time permuting rows and columns in the same manner, as is standard procedure in Mantel tests. An overview of dissimilarities and geographic distances is shown in Fig. S5.

To examine the potential ability of environmental variables to explain taxonomic composition differences between samples, we attempted to build linear regression models, whose response variables were pairwise dissimilarities (Bray-Curtis or Jaccard or Hellinger) in taxonomic composition (at ASV level, or OTU level etc) and whose potential predictor variables were pairwise geographic distances as well as pairwise absolute differences in the 20 physical-chemical variables mentioned earlier (regional environmental variables, ICP-OES measurements, in-situ salinity). Hence, each sample pair constituted a single datapoint for the model. Linear coefficients were fitted using least squares, and predictors were added one at a time using a stepwise approach whereby a predictor was only included if its linear coefficient was statistically significantly different from zero (*P* < 0.05). This statistical significance was assessed through simultaneous permutations of the response matrix’s rows and columns, similar to the permutation null models commonly deployed in Mantel tests, following Legendre et al.^65^.

### Analysis of taxon co-distributions

To examine potential interdependencies between taxon distributions across samples, we performed two alternative null hypothesis tests separately for each taxonomic level (ASVs, OTUs, genera etc). Both tests are commonly used in ecology to detect non-neutral patterns in the joint distributions of multiple species, for example resulting from competitive exclusion or mutualisms^50^. Each test defines a test statistic that conceptually corresponds to a notion of taxon overlaps, or a correlation in the distribution of taxa, as well as a null model for generating random data for computing statistical significances. In the first test, we considered a summary statistic computed based on the presences/absences of taxa in each sample, henceforth referred to as *checkerboard cooccurrence* (CC) score:

1 $${{\rm{CC}}}=1-\frac{\mathop{\sum }_{i = 1}^{M}\mathop{\sum }_{j = 1}^{i-1}({N}_{i}-{N}_{ij})({N}_{j}-{N}_{ji})}{\mathop{\sum }_{i = 1}^{M}\mathop{\sum }_{j = 1}^{i-1}({N}_{i}-N{p}_{i}{p}_{j})({N}_{j}-N{p}_{i}{p}_{j})}$$

where *M* is the total number of considered taxa, *N* is the total number of samples, *N*_*i*_ is the number of samples containing the *i*’th taxon, *N*_*ij*_ is the number of samples containing both taxa *i* and *j* and *p*_*i*_: = *N*_*i*_/*N*. Hence, for fixed *N*_1_, *N*_2_, . . , *N*_*M*_, the CC-score becomes larger if taxa co-occur more frequently (i.e., *N*_*ij*_ are larger). Note that this summary statistic is closely related to the “C score” described by Gotelli^50^, with two differences: The CC score is normalized differently such that its scale remains roughly constant as the number of samples increases, and it is reversed such that a greater value corresponds to a greater overlap in taxon distributions. To assess whether an observed CC score was probably due to chance (i.e., if taxa occur independently of each other), we compared it to the CC score distribution of 1000 presence-absence matrices randomly generated under a null model. As null we used the “SIM9” permutation model described by Gotelli^50^, also known as “fixed-fixed” model because it preserves the number of taxa per sample and the number of samples per taxon^31,66^. If random CC scores generated by the null model are mostly above the observed CC score, this would mean that taxa tend to exclude each other more often than expected by chance (i.e., taxa are segregated). To account for multiple hypothesis tests (i.e., one for each taxonomic level), we also considered a Bonferroni-adjusted significance threshold of $\tilde{\alpha }=\alpha /n=0.05/7=0.0071$. An overview of results is shown in Table S5.

In the second test, we considered a summary statistic based on the relative abundances of taxa in each sample, known as *generalized Morisita similarity index*^67^ and henceforth referred to as “MA-score”^51^:

2 $${{\rm{MA}}}=\frac{\mathop{\sum }_{i = 1}^{M}\left[{\left(\mathop{\sum }_{j = 1}^{N}{p}_{ij}\right)}^{2}-\mathop{\sum }_{j = 1}^{S}{p}_{ij}^{2}\right]}{(N-1)\mathop{\sum }_{i = 1}^{M}\mathop{\sum }_{j = 1}^{N}{p}_{ij}^{2}},$$

where *p*_*ij*_ is the relative abundance of taxon *i* in sample *j*. Hence, a lower MA score indicates a lower similarity between samples in terms of taxon abundances and thus a potential segregation between taxa. As null we considered the “IT” model suggested by Ulrich et al.^51^, which assigns reads to matrix cells proportional to the total number of reads in each sample and proportional to the total number of reads assigned to each taxon across samples, until the total number of reads per sample and per taxon is reached^31^. We used 1000 abundance matrices randomly generated by this model to asses the statistical significance of MA scores.

### Statistics and reproducibility

Unless mentioned otherwise, all statistical analyses involved the 10 independent and unique microbiome samples described above. All statistical analyses can be reproduced following the details described above.

### Reporting summary

Further information on research design is available in the Nature Portfolio Reporting Summary linked to this article.

## Supplementary information

Supplemental material

Description of Additional Supplementary Files

Supplementary Data 1

Supplementary Data 2

Supplementary Data 3

Reporting summary

## Data Availability

Raw metagenomic and amplicon sequence data are available on the NCBI Sequence Read Archive under BioProject PRJNA1114803, BioSamples SAMN41500170–SAMN41500179, runs SRR29139261–SRR29139270 (metagenomes) and SRR29138647–SRR29138656 (16S rRNA gene amplicons). Sample metadata, including measured environmental conditions, are available as Supplementary Data 1. Metagenomic gene profiles (abundances per sample) are provided as Supplementary Data 2. Taxonomic profiles are provided as Supplementary Data 3. All other data are available from the corresponding author on reasonable request.

## Abbreviations

ASV: amplicon sequence variant
OTU: operational taxonomic unit
CV: coefficient of variation

## Code availability

All software used in this paper have been described in the Methods and are freely available online.
