# Supplementary material for: Taxonomic variability and functional stability across Oregon coastal subsurface microbiomes
Source: Commun Biol. 2024 Dec 19;7:1663. doi: 10.1038/s42003-024-07384-y (PMC11659426; doi:10.1038/s42003-024-07384-y)
Supplement: Supplementary file 1 — Supplemental material [file 42003_2024_7384_MOESM1_ESM.pdf]

Taxonomic variability and functional stability across Oregon coastal subsurface microbiomes
# - Supplementary Information -

Table S1: **Sample summaries.** Overview of sampling locations, sampling dates and numbers of raw metagenomic and 16S rRNA gene amplicon reads. For additional sample metadata see Supplemental File 1.

sample	nearest delta	date (Y.M.D)	latitude	longitude	metag. reads	16S reads
ColS0	Columbia	2023.10.14	46.224290	-124.009156	2979797	10499
CsN0	Coos	2023.10.21	43.357270	-124.338938	6455597	18295
CsN2	Coos	2023.10.21	43.372205	-124.326941	3958666	18975
CsN4	Coos	2023.10.21	43.389272	-124.314713	4492886	6910
NN35	Nehalem	2023.11.11	45.689080	-123.940613	5319442	6113
NN5	Nehalem	2023.11.11	45.703327	-123.941195	4210128	11504
SilS5	Siletz	2023.11.12	44.882809	-124.036913	5810252	9234
SiuS0	Siuslaw	2023.10.08	44.016103	-124.138902	6891881	25207
SiuS2	Siuslaw	2023.10.08	43.998559	-124.138189	5555930	10920
SiuS4	Siuslaw	2023.10.08	43.980818	-124.140290	2209106	801

Table S2: **River summaries.** Overview of the major river deltas near the sampling locations.

delta	typical discharge rate	origin
Columbia	5500 $m^3 \cdot s^{-1}$ [1]	Rocky Mountains of BC, Canada
Coos	41 $m^3 \cdot s^{-1}$ [2]	South-West Oregon
Nehalem	75 $m^3 \cdot s^{-1}$ [3]	Northern Oregon coast range
Siletz	42 $m^3 \cdot s^{-1}$ [4]	Central Oregon coast range
Siuslaw	56 $m^3 \cdot s^{-1}$ [5]	Central Oregon coast range

Table S3: **Mantel tests (taxonomic dissimilarities vs geographic distances).** Overview of Mantel tests of Spearman rank correlations, at each taxonomic level and for each of the three considered dissimilarity metrics. The table lists Spearman correlation coefficients (ρ) and associated one-sided statistical significances (P). Significances below 0.05 are bolded. Adjusting the significance threshold to account for multiple hypothesis tests using a Bonferroni correction ($\alpha = 0.05/21 = 0.0024$) renders all Mantel tests statistically insignificant. All tests compared 10 samples (45 unique pairs).

	**Jaccard**		**Bray-Curtis**		**Hellinger**	
**tax. level**	ρ	P	ρ	P	ρ	P
phylum	0.16	0.15	-0.14	0.17	0.35	**0.021**
class	0.15	0.16	-0.081	0.33	0.25	0.071
order	0.16	0.16	-0.07	0.35	0.18	0.11
family	0.16	0.15	-0.06	0.41	0.19	0.12
genus	0.12	0.19	-0.043	0.46	0.26	0.058
OTU	0.088	0.27	0.025	0.38	0.08	0.23
ASV	0.044	0.35	0.055	0.33	0.066	0.31

Table S4: **Regression models.** Overview of regression model selection, predicting pairwise taxonomic dissimilarities (Jaccard, Bray-Curtis or Hellinger) as a function of pairwise absolute differences in environmental variables and/or geographic distances. The table includes the predictors selected at each taxonomic level and associated P-values in parentheses, as well as the fraction of variance explained by the models based on leave-one-out cross validation (R^2_{cv}). Note that in most cases no predictors were selected, i.e., none had a statistically significant coefficient. In fact, if the significance threshold were to be adjusted for multiple hypothesis tests ($\alpha = 0.05/(3 \cdot 7 \cdot 21) = 0.00011$), then no predictor would have been selected in any case.

	**Jaccard**		**Bray-Curtis**		**Hellinger**	
**tax. level**	**predictors**	R^2_{cv}	**predictors**	R^2_{cv}	**predictors**	R^2_{cv}
phylum	-	-	-	-	Boron (P=0.042)	0.27
class	-	-	-	-	Boron (P=0.036)	0.26
order	-	-	-	-	-	-
family	-	-	-	-	Boron (P=0.033)	0.22
genus	-	-	-	-	Boron (P=0.023)	0.24
OTU	-	-	-	-	-	-
ASV	-	-	-	-	-	-

Table S5: **Cooccurrence analysis (CC scores).** Overview of checkerboard cooccurrence (CC) analyses across samples, at various taxonomic levels. The table shows the number of taxa analyzed, the CC score of taxon presences/absences, the mean CC score under the null model (i.e., expectation under independent taxon distributions), the standardized effect size (SES) and the two-sided statistical significance (probability that the null model would generate a more extreme SES than observed). Note that a lower CC score implies a lower overlap of taxon distributions. Taxonomic levels where the CC score was significantly different from the null model's expectation (P<0.05) are bolded. Taxonomic levels for which P falls below the Bonferroni-corrected significance threshold (P<0.0071), i.e., accounting for 7 independent hypothesis tests, are underlined.

tax. level	Ntaxa	CC score	mean null	SES	significance (P)
phylum	34	0.623	0.635	-0.53	0.563
**class**	75	0.624	0.659	-2.60	0.011
**<u>order</u>**	130	0.443	0.489	-6.14	<0.001
**<u>family</u>**	155	0.392	0.441	-7.41	<0.001
**<u>genus</u>**	193	0.397	0.450	-10.5	<0.001
**<u>OTU</u>**	1507	0.275	0.311	-32.1	<0.001
**<u>ASV</u>**	4315	0.234	0.253	-36.1	<0.001

Table S6: **Coabundance analysis (MA scores).** Overview of the Morisita coabundance analyses across samples, at various taxonomic levels. The table shows the number of taxa analyzed, the MA score of relative taxon abundances, the mean MA score under the null model (i.e., expectation under independent taxon distributions), the standardized effect size (SES) and the two-sided statistical significance (probability that the null model would generate a more extreme SES than observed). Note that a lower MA score implies a lower overlap of taxon distributions. At all taxonomic levels, MA scores were significantly lower than the null model's expectation (P<0.05), even after a Bonferroni correction accounting for multiple hypothesis tests (P<0.0071).

tax. level	Ntaxa	MA score	mean null	SES	significance (P)
phylum	34	0.922	0.998	-81.6	<0.001
class	75	0.896	0.996	-79.8	<0.001
order	130	0.872	0.994	-69.6	<0.001
family	155	0.884	0.994	-62.4	<0.001
genus	193	0.830	0.984	-47.7	<0.001
OTU	1507	0.459	0.844	-60.0	<0.001
ASV	4315	0.225	0.472	-44.8	<0.001

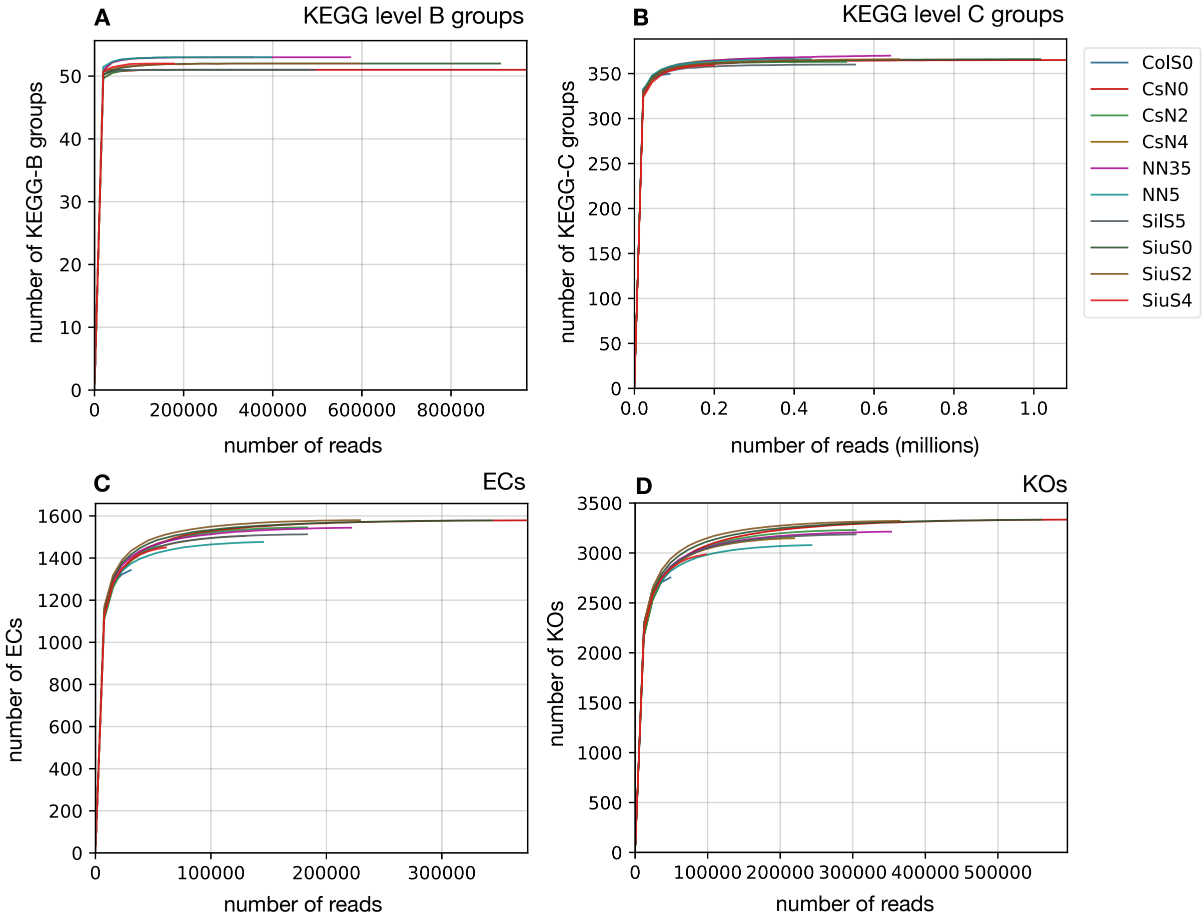

Figure S1: **Collector's curves (KOs vs. reads).** (A) Collectors curves, showing for each sample the expected number of gene groups (KEGG level B) discovered as a function of the number of reads. Each curve was computed by repeatedly randomly subsampling (rarefying) reads in a sample, counting the number of gene groups represented by the retained reads, and averaging over all repeats. (B) Similar to A, but for KEGG level C groups. (C) Similar to A, but with KOs grouped according to their Enzyme Commission (EC) numbers. (D) Similar to A, but for individual KEGG orthologs (KOs).

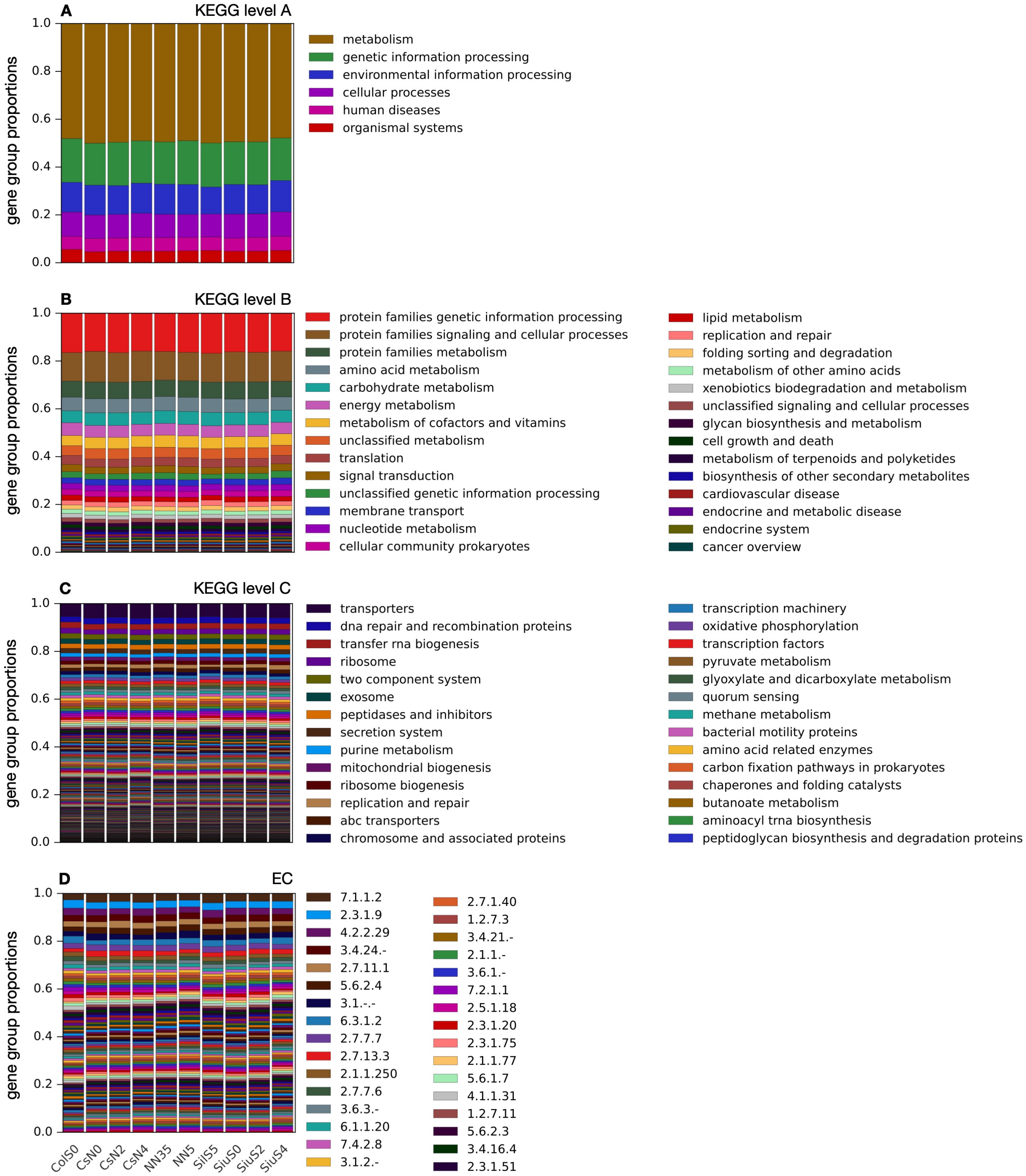

Figure S2: **Gene group profiles (all KEGG levels).** Estimated proportions (relative abundances) of genes associated with various standardized KEGG categories, at hierarchical levels A, B and C, as well as various Enzyme Commission (EC) numbers. Proportions are based on the average number of metagenomic reads mapped per protein basepair. Proportions are normalized in each sample such that their sum over all gene groups is 1. Only the most abundant gene groups are listed in the legends for simplicity. In (D) only the 100 most abundant ECs are shown for visibility.

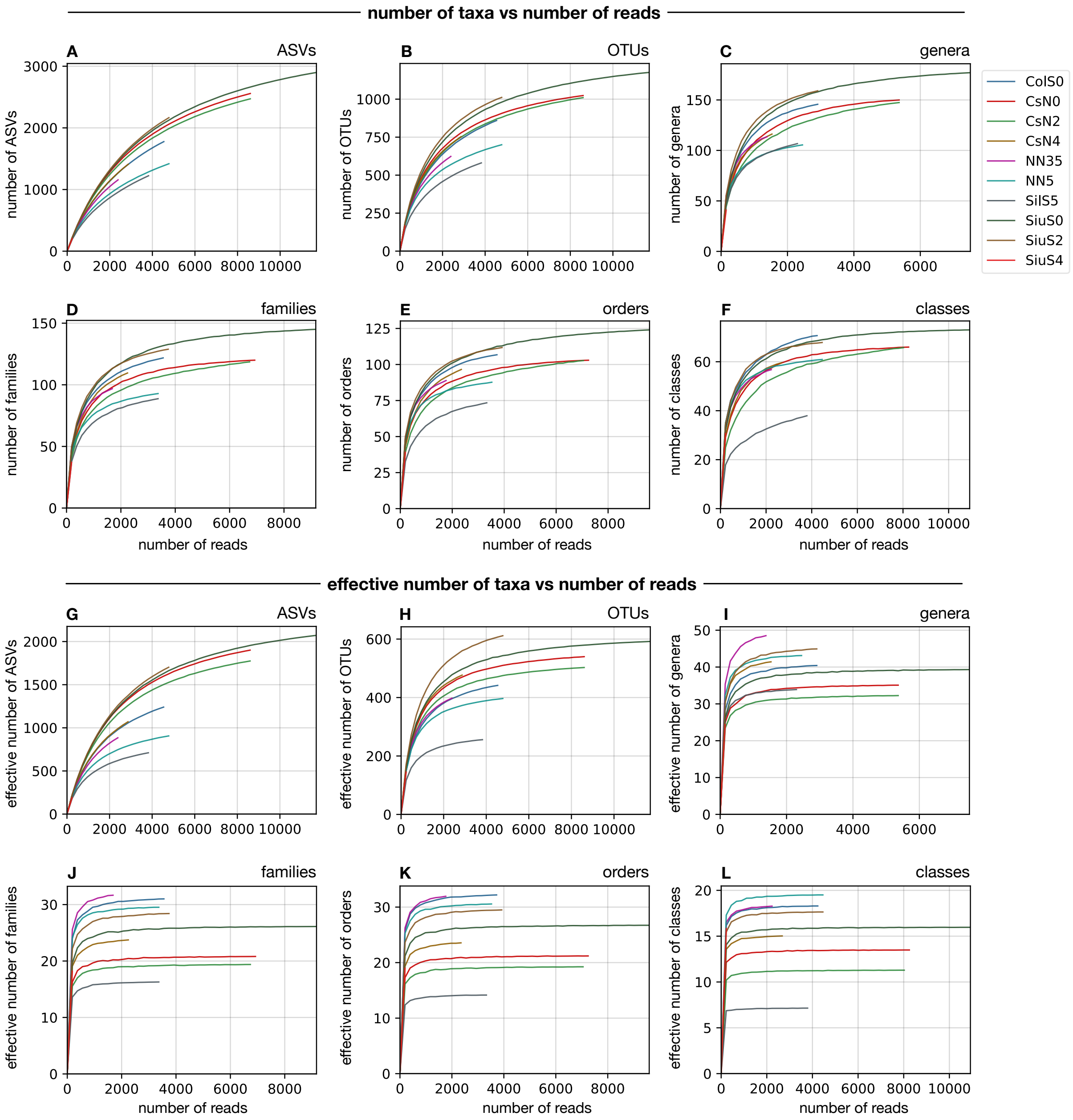

Figure S3: **Collector's curves (taxa vs. reads).** (A) Collectors curves, showing for each sample the expected number of ASVs discovered as a function of the number of reads. Each curve was computed by repeatedly randomly subsampling (rarefying) reads in a sample, counting the number of ASVs represented by the retained reads, and averaging over all repeats. (B–F) Similar to A, but for OTUs, genera, families, orders and classes. (G–L) Similar to A–F, but instead showing the *effective* number of taxa based on Shannon diversities, i.e. the expected hypothetical number of equally abundant taxa that would yield the same Shannon diversity as observed.

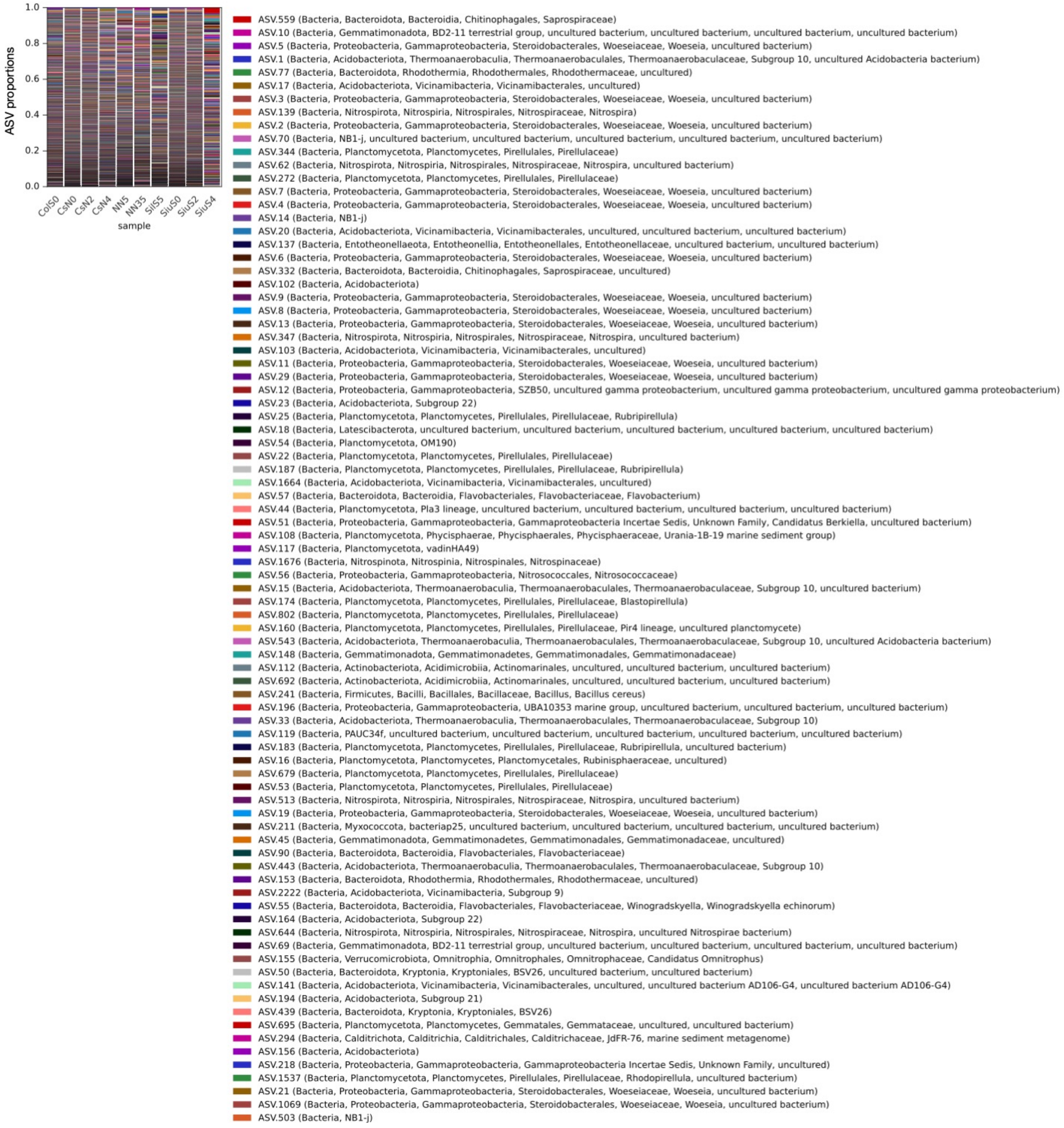

Figure S4: **ASV proportions.** Proportions (relative abundances) of 16S rRNA gene amplicon sequence variants (ASVs) detected in all samples, based on the number of reads mapped to each ASV. ASVs are sorted from top to bottom in decreasing average proportion. Only the most abundant ASVs are listed in the legend for readability. Estimated taxonomic identities are shown in parentheses. While archaeal ASVs were also detected, they were relatively rare and are thus not listed in the legend.

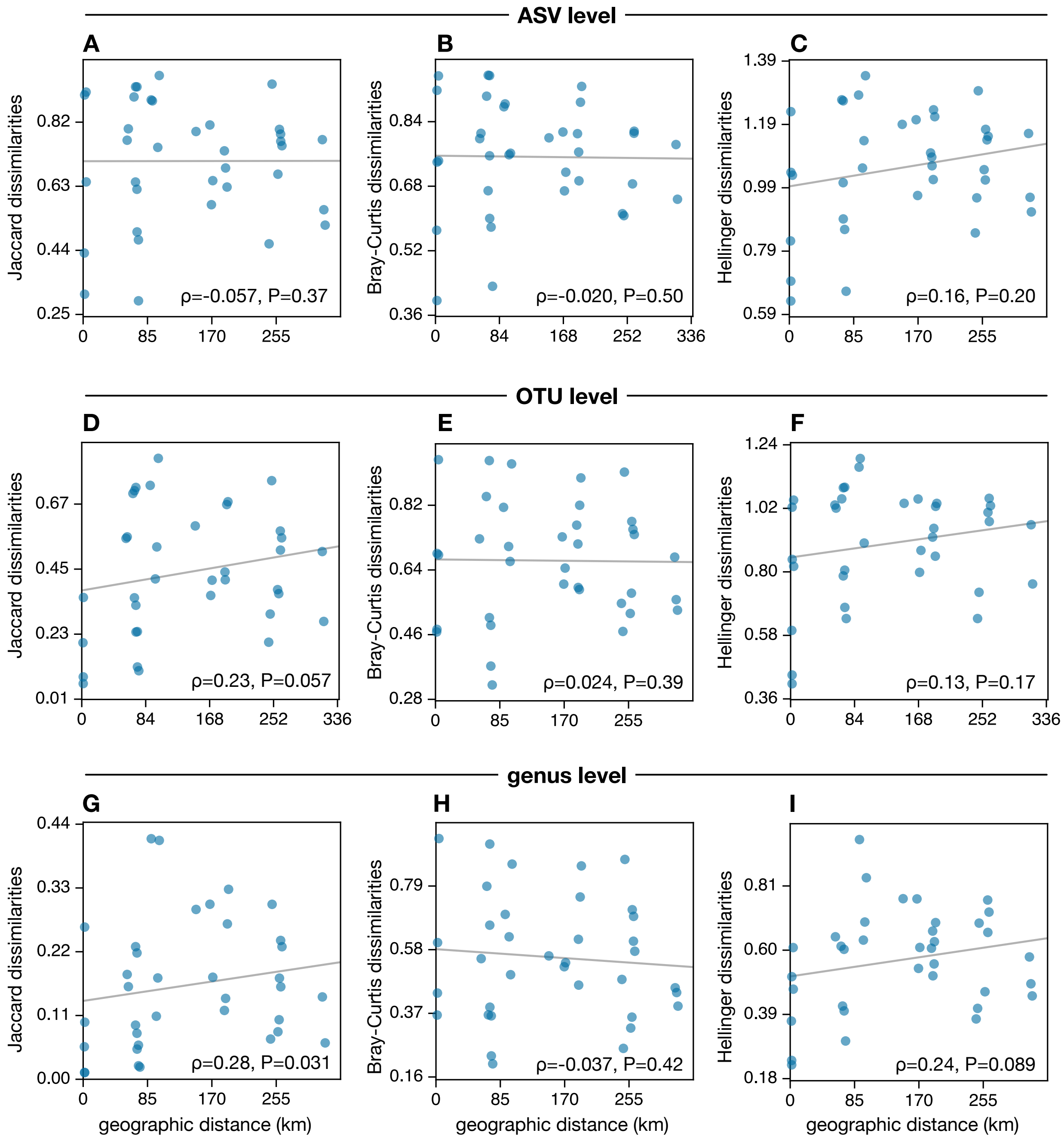

Figure S5: **Mantel tests.** (A) Pairwise Jaccard dissimilarities between samples at the ASV level (vertical axis) compared to pairwise geographic distances (horizontal axis). Each point represents a distinct pair of samples. A linear regression line (fitted via least squares) is shown for reference. The Spearman rank correlation (ρ) and its statistical significance (P), assessed via a Mantel test (random permutations of the rows and columns of the dissimilarity matrix), are shown inside the figure. (B,C) Similar to A, but considering the Bray-Curtis and Hellinger dissimilarity metrics. (D–F) Similar to A–C, but evaluating dissimilarities at the level of OTUs. (G–I) Similar to A–C, but evaluating dissimilarities at the level of genera. For additional taxonomic levels see Supplemental Table S3.

References

[1] Lutz, G., Hubbell, D., Stevens, H., (U.S.), G.S. & Commission, U.A.E. *Discharge and flow distribution, Columbia River estuary*. No. v. 433 in Discharge and Flow Distribution, Columbia River Estuary (U.S. Government Printing Office, 1975).

[2] Palmer, T. & Avery, W. *Field guide to Oregon rivers* (Oregon State University Press, 2014).

[3] Water-data report 2007: 14301000 Nehalem River near Foss, OR. Tech. Rep., United States Geological Survey (2007).

[4] Water-Data Report 2010: 14305500 Siletz River at Siletz, OR. Tech. Rep., United States Geological Survey (2010).

[5] Water-Data Report 2010: 14307620 Siuslaw River near Mapleton, OR. Tech. Rep., United States Geological Survey (2010).
