# Supplementary material for: Taxonomic variability and functional stability across Oregon coastal subsurface microbiomes
Source: Commun Biol. 2024 Dec 19;7:1663. doi: 10.1038/s42003-024-07384-y (PMC11659426; doi:10.1038/s42003-024-07384-y)
Supplement: Supplementary file 2 — Description of Additional Supplementary Files [file 42003_2024_7384_MOESM2_ESM.pdf]

Description of Additional Supplementary Files

File name: Supplementary Data 1
Description: Sample metadata

File name: Supplementary Data 2
Description: Gene profiles

File name: Supplementary Data 3
Description: 16S OTU, ASV and taxon tables
